# In-line milk progesterone monitoring as a tool for precision reproductive management

**DOI:** 10.3168/jdsc.2024-0649

**Published:** 2024-10-29

**Authors:** Tony C. Bruinjé, Divakar J. Ambrose, Stephen J. LeBlanc

**Affiliations:** 1 Department of Population Medicine, University of Guelph, Guelph, ON, Canada N1G 2W1; 2 Department of Agricultural, Food and Nutritional Sciences, University of Alberta, Edmonton, AB, Canada T6G 2P5

## Abstract

In-line milk progesterone (IMP4) monitoring (Herd Navigator, DeLaval) is a technology that automatically detects onset of cyclicity, estrus, and pregnancy. Sampling starts at 20 DIM and occurs on average every 2 d until pregnancy. Estrus is detected based on a decline in progesterone (P4) concentrations below a threshold, and pregnancy is assessed from 30 to 55 d after AI in cows without return to estrus. Here, we review the potential of IMP4 as a tool for reproductive management. In a series of observational studies with up to 158,961 IMP4 records from 4,353 AI events, we characterized predictors of pregnancy per AI (P/AI) and investigated IMP4 profiles in cows returning to estrus. Some of the predictors included prolonged luteal phase before AI and suboptimal P4 levels at different time points before and after AI. Over one-third of cows had at least one characteristic of P4 profile unfavorable to P/AI, but with low predictive abilities. Among nonpregnant cows, 5% returned to estrus by 17 d after AI, 64% between 18 and 24 d, 16% between 25 and 30 d, and 15% between 31 and 55 d. This represents 85% of cows that are not pregnant 55 d after AI returning to estrus before 30 d, when first pregnancy diagnosis occurs in many dairies. Monitoring IMP4 might be used to identify subgroups of cows with different predicted P/AI to develop selective breeding strategies or targeted interventions. It can also aid in identifying nonpregnant cows early for timely reinsemination.

## Highlights

• In-line milk progesterone can detect cyclicity, pregnancy, and return to estrus.
• Characteristics of IMP4 before and after AI can be used as predictors of P/AI.
• 85% of nonpregnant cows were identified as returning to estrus before 30 days after AI.
• IMP4 might be used to develop selective interventions and aid with early reinsemination.

## Graphical Abstract

**Figure:**
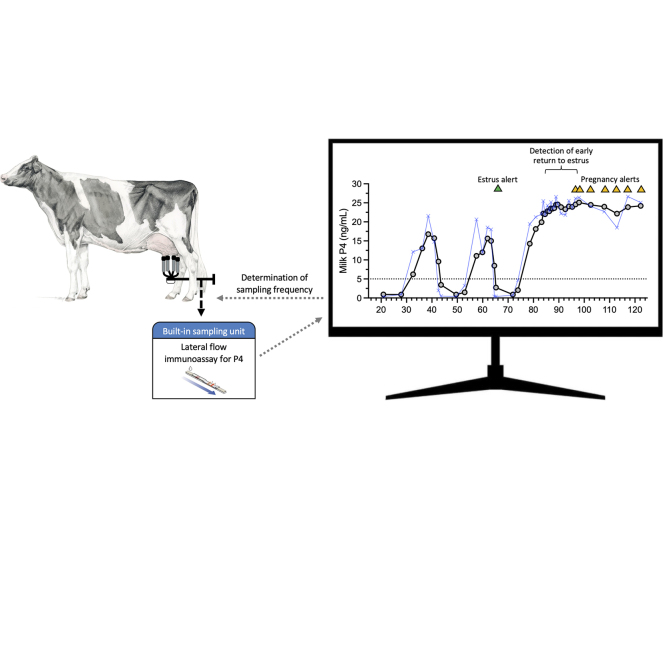
**Summary:** In-line milk progesterone (IMP4) monitoring automatically samples and measures milk progesterone (P4) and detects the onset of cyclicity, estrus, and pregnancy status. Recent studies characterized markers of IMP4 before and after artificial insemination (AI) associated with pregnancy failure, such as prolonged luteal phase before AI and suboptimal P4 levels at different time points before and after AI. Among cows returning to estrus by 55 days after AI, IMP4 detected 69% of cows returning to estrus before 24 days, 16% between 25 and 30 days, and 15% between 31 and 55 days. This means that 85% of cows that were not pregnant at 55 days could be detected returning to estrus before 30 days, which is the typical time of first pregnancy diagnosis at many dairies. Monitoring IMP4 can be used to identify cows with different predicted probabilities of pregnancy per AI and to detect early return to estrus for timely reinsemination.

Reproductive monitoring technologies for estrus detection were developed over 20 yr ago, but newer sensor technologies have become more popular over the last 10 to 15 yr (Saint-Dizier and Chastant-Maillard, 2012; Fricke et al., 2014). The growing popularity of automated estrus detection devices, such as activity monitors, can be attributed to their high estrus detection rates (≥70%; Saint-Dizier and Chastant-Maillard, 2012; Bruinjé and LeBlanc, 2024), as well as their potential to optimize labor efficiency and integrate into reproductive management programs (Giordano et al., 2022; Bruinjé and LeBlanc, 2024). However, there is limited literature about the application of an automated in-line milk progesterone (**IMP4**) technology for monitoring reproductive function and supporting reproductive management. This brief narrative review summarizes recent research on that.

An IMP4 tool (Herd Navigator, DeLaval) has been commercially available in Europe and Canada since the early 2010s and is currently available in 4 states in the United States. The current IMP4 sensor is a module attached to automated milking systems that automatically samples ∼50 mL of milk during milking and quantifies progesterone (**P4**) concentration (∼0 to 28 ng/mL) using a lateral flow immunoassay dry-stick technology. Samples are taken and analyzed at frequent intervals determined by a biomodel according to the stage of the estrous cycle: postpartum anestrous, estrous cycling, or potentially pregnant (Friggens and Chagunda, 2005), as illustrated in Figure 1. The estrous cycle events are indicated based on fluctuations in adjusted milk P4, determined by proprietary algorithms, above or below a threshold of 5 ng/mL that is indicative of luteal activity (Adriaens et al., 2019). The biomodel uses each estrus event as a reference point for subsequent sampling time points to detect return to estrus or pregnancy. As a result, sampling time points following spontaneous estrus are more accurate compared with those following synchronized estrus. If the cow receives AI and does not return to estrus by 32 d after, the cow is classified as potentially pregnant. This biomodel was validated to detect estrus with 93% sensitivity (**Sn**) and 94% specificity (**Sp**; Friggens et al., 2008). However, few studies have explored the further potential of using this technology for precision reproductive management.

**Figure 1:**
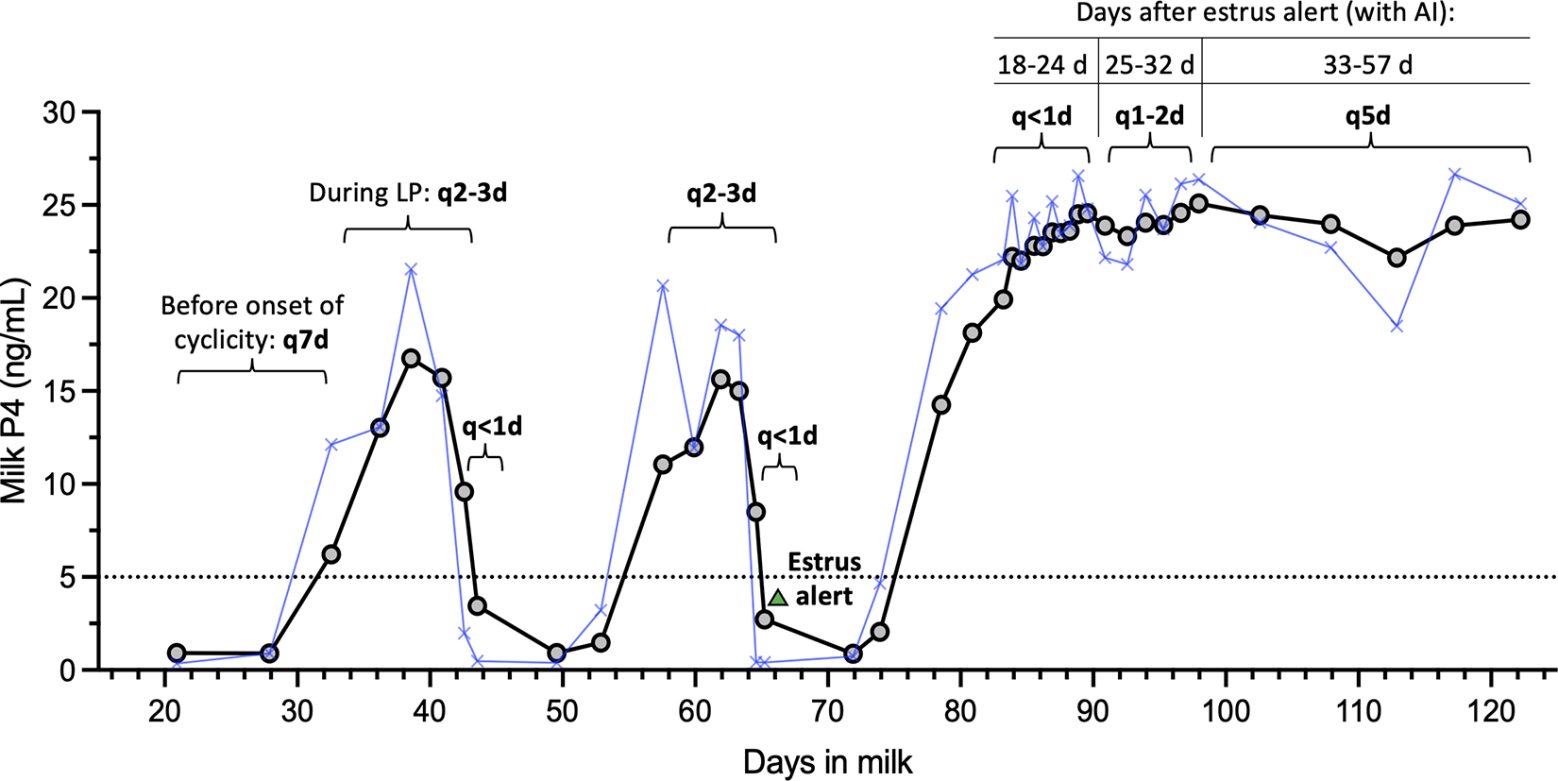
Frequency of samples automatically taken and analyzed by the in-line milk progesterone (IMP4) monitoring (Herd Navigator, DeLaval) biomodel. The blue symbols represent raw (unadjusted) milk progesterone concentrations, and the black/gray symbols represent adjusted concentrations. Sampling frequency varies according to the estimated stage of the estrous cycle (postpartum anestrous, estrous cycling, or potentially pregnant) and increases when an estrus event (decline in IMP4 below 5 ng/mL) is expected. Briefly, sampling occurs every 7 d (q7d) until onset of cyclicity, then every 2 to 3 d (q2–3d) during luteal phases and then once or twice daily (q<1d) when estrus is expected. If the cow receives AI at estrus and does no return to estrus until 24 d, sampling occurs every 1 to 2 d (q1–2d) until 32 d and every 5 d (q5d) until 57 d (Bruinjé et al., 2019).

In a series of observational studies with cows bred based on IMP4 estrus alerts and no hormonal interventions, we investigated whether IMP4 profiles before and after AI were associated with the likelihood of pregnancy (Bruinjé et al., 2017a,b, 2019). Primiparous cows with onset of cyclicity by 28 DIM (21% prevalence) had greater pregnancy per AI (**P/AI**) at first AI (47% vs. 32%) than cows with later cyclicity. For multiparous cows, lower P/AI (7% vs. 29%) and greater presumed pregnancy loss (return to estrus between 30 and 55 d after AI; 69% vs. 35%) were observed in cows with delayed onset of cyclicity (>56 DIM; 14% prevalence) compared with cows with earlier cyclicity. During the voluntary waiting period (**VWP**), 48% of primiparous and 54% of multiparous cows had at least one abnormal LP, defined as either <7 d or ≥19 d long (Bruinjé et al., 2017a). Although the high prevalence of abnormal LP could have been attributed to the arbitrary criterion used for short or prolonged LP, primiparous cows with at least one abnormal LP had lower P/AI at first AI (30% vs. 40% at 30 d) and greater pregnancy loss (36% vs. 23% returning to estrus between 30 and 55 d) compared with those with normal LP. Cows that became pregnant had greater milk P4 concentrations from 10 to 21 d after AI than those that returned to estrus by d 30 (Bruinjé et al., 2017b). Pregnant multiparous cows had slightly lesser milk P4 at estrus alert (3.2 vs. 3.4 ng/mL) and had a greater increase in milk P4 from d 5 to 14 than cows that returned to estrus. Similarly, others reported that cows returning to estrus between 25 and 41 d had lesser milk P4 at d 10 and 20 than pregnant cows (Ask-Gullstrand et al., 2021). Although the statistical power in some of these studies was limited, particularly for pregnancy loss, they suggest that IMP4 data during the VWP or around time of AI can be useful in identifying cows with different probable reproductive outcomes.

In a subsequent study (Bruinjé et al., 2019), we evaluated 158,961 IMP4 records and 4,353 AI events that occurred based on estrus alerts by IMP4 from 1,891 lactations in 4 herds. Markers of IMP4 profiles, such as LP length and peak P4 preceding estrus, nadir P4 at time of estrus, interval from AI to onset of subsequent LP, and P4 concentrations after AI were characterized (Figure 2). There were significant linear or quadratic associations of all the above with P/AI. However, the predicted associations varied considerably by herd and other covariates, resulting in low accuracy of classification (receiver operating characteristic area under the curve <0.60). The variation in P/AI was largely attributed to different herds, with average herd-specific P/AI varying from 19% to 50%. Additional sources of variation included in the prediction models were parity (average of 35%, 34%, and 30% P/AI in first, second, and third or greater lactations, respectively) and year (36%, 33%, and 29% in 2014, 2015, and 2016, respectively). Still, cows classified in the unfavorable categories of P4 profiles had significantly lesser predicted P/AI than cows in the favorable categories. The prevalences of unfavorable IMP4 markers were high, with 31% prolonged LP, 46% low peak P4 before AI, 42% elevated nadir P4, and 28% and 51% low P4 at d 10 and 14, respectively. Although the ability of the cutpoints to discriminate categories with different predicted P/AI were low, they could be used to identify subgroups of cows at risk of lower P/AI that might benefit from targeted interventions.

**Figure 2:**
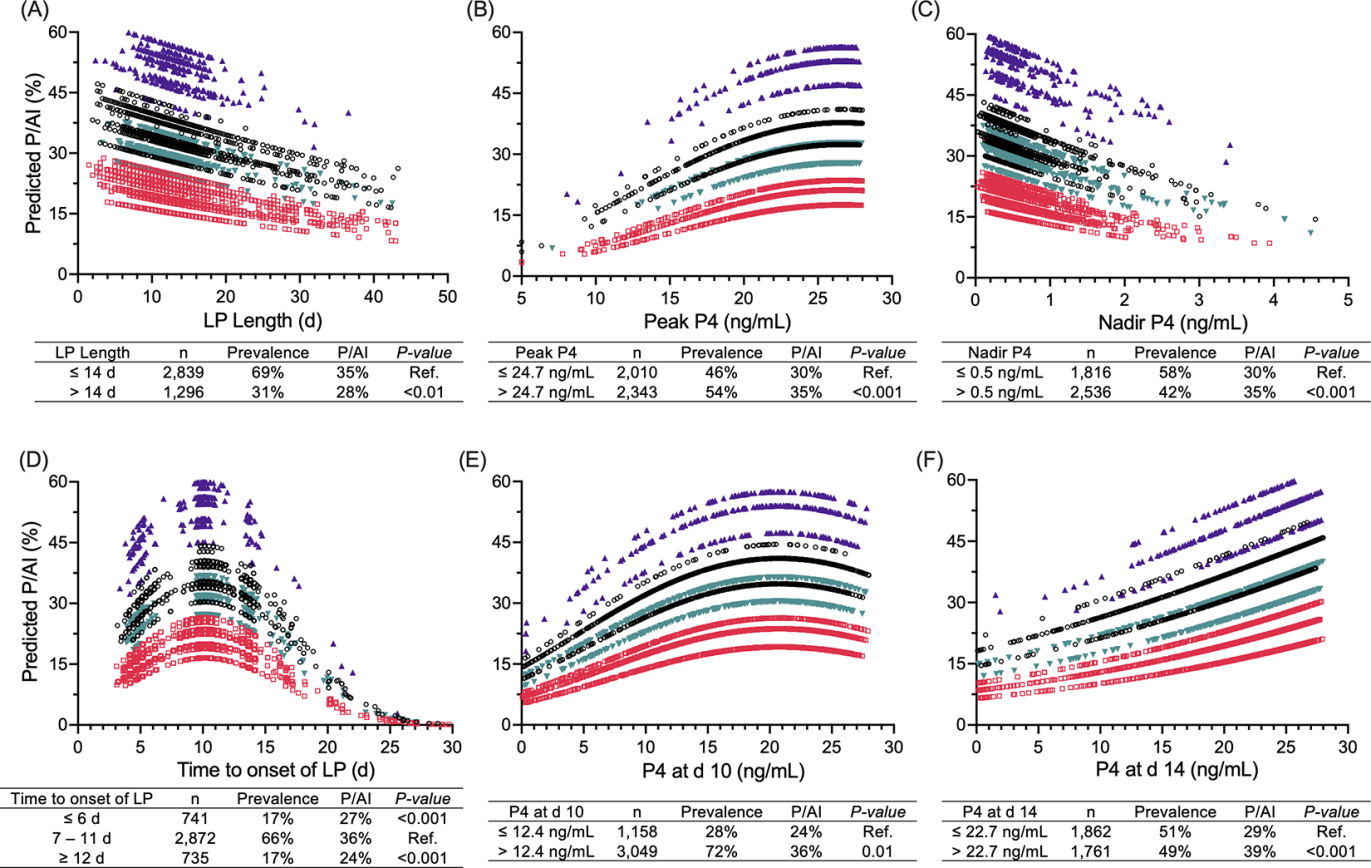
Associations of IMP4 variables obtained before AI (panels A–C) and after AI (panels D–F) with the predicted pregnancy per AI (P/AI) in 4,353 AI events in 4 herds, as described elsewhere (Bruinjé et al., 2019). The variation in the associations was attributed to different herds (each represented in a different color), and other covariates in multivariable models. Tables show the comparisons between categories defined based on receiver operating characteristic cutpoints (panels A–C, E, F) or based on sampling frequency (panel D). P4 = progesterone; LP = luteal phase; peak P4 = maximum P4 in the LP preceding AI; nadir P4 = P4 at onset of estrus; time to onset of LP = interval to increase in P4 to ≥5 ng/mL after AI. Ref. = referent.

The accuracy of pregnancy alerts generated by IMP4 from 30 to 55 d after AI in cows not returning to estrus was evaluated following 1,821 AI (Bruinjé and Ambrose, 2019). The Sn and Sp to determine pregnancy was 95% and 86% at 30 d after AI, 96% and 89% at 37 d, 97% and 94% at 44 d, and 97% and 98% at 51 d (±3), respectively. The increasing Sp over time was expected as cows are less likely to return to estrus and be false positives (e.g., due to embryonic losses) at later stages of pregnancy.

Here, we evaluated data on return to estrus after 4,353 inseminated estrus events from a dataset used in Bruinjé et al. (2019). On average (±SD), AI occurred 1.9 ± 0.5 d after the estrus alert. Among the nonpregnant cows at 55 d after AI (74% of all), 5% returned to estrus before 17 d after AI (4% of all), 64% between 18 and 24 d (46% of all), 16% between 25 and 30 d (12% of all), 8% between 31 and 40 d (6% of all), and 7% between 41 and 55 d (5% of all) (Figure 3). In another study with 5,820 AI events, 80% had return to estrus before 24 d, 12% between 25 and 41 d, and 8% after 41 d (Ask-Gullstrand et al., 2021). In summary, about 85% of cows that are not pregnant at 55 d after AI may be detected by IMP4 returning to estrus before 30 d, when pregnancy alerts initiate. It also indicates that up to one-third of nonpregnant cows may return to estrus beyond the expected interval of 18 to 24 d.

**Figure 3:**
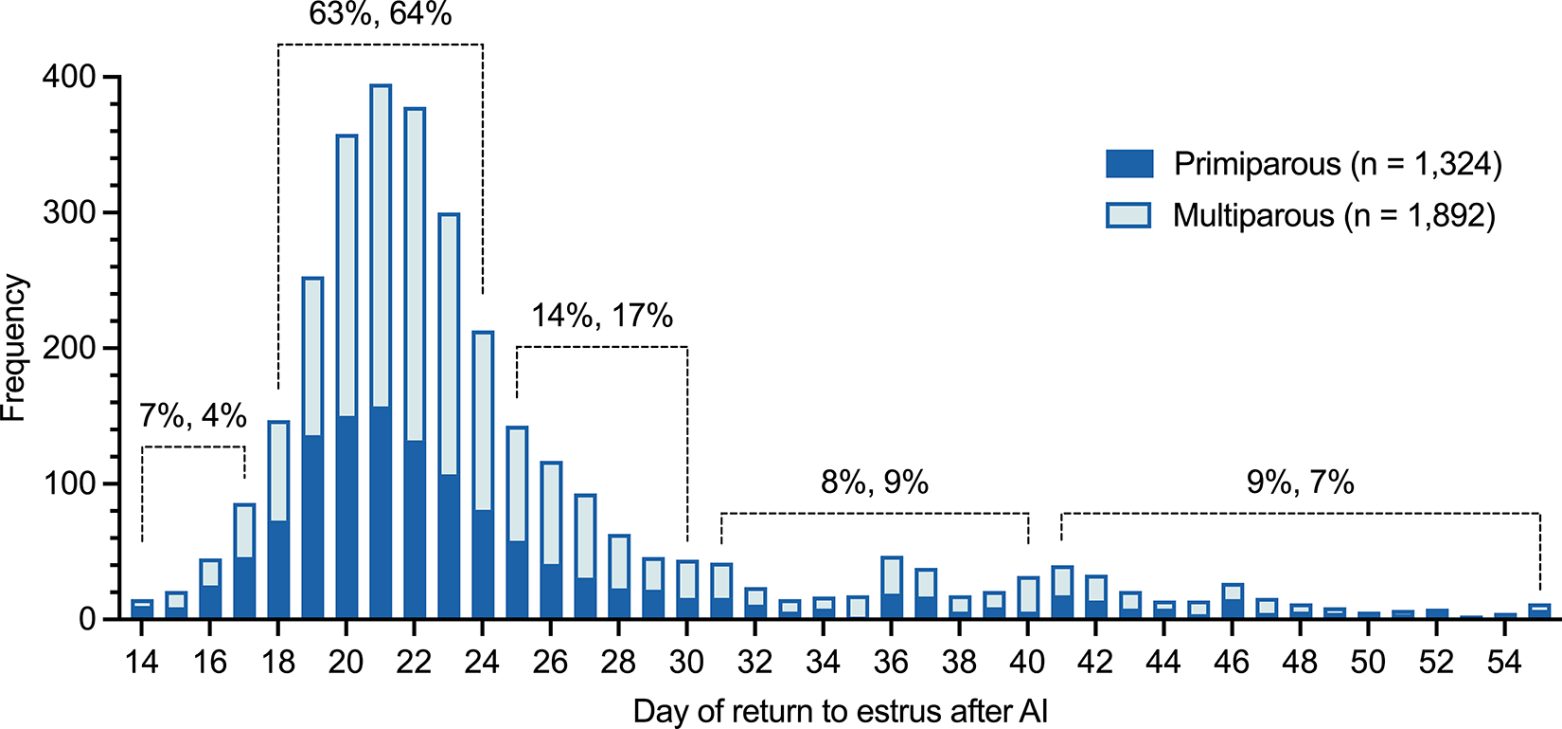
Distribution of time to return to estrus following 3,218 AI events in cows that did not result in a pregnancy by 55 d, from a total of 4,353 AI events, from 4 dairy herds in a dataset described in Bruinjé et al. (2019). Estrus events immediately preceding AI and at return to estrus were detected by IMP4 monitoring (Herd Navigator, DeLaval). The proportions above each category are of primiparous and multiparous cows, respectively.

Estrous cycle length has been estimated in most studies based on interservice interval, milk P4, or corpus luteum (**CL**) ultrasound. In a dataset of >114,000 interservice intervals, 59% were between 18 and 24 d, among those <30 d long (Remnant et al., 2015). Blavy et al. (2016) reported that 40% of 1,400 estrous cycles evaluated by milk P4 were >24 d long. These estimates were greater than in a previous study with 137 cows where 26% had a LP >24 d long (Ricci et al., 2017). Among the 80 nonpregnant cows (confirmed on d 32), 55% had CL regression between 18 and 22 d, 23% between 25 and 32 d, and 21% maintained the CL until d 32. Although datasets were different, the consistently greater than expected proportion of cows with an extended interservice interval or late return to estrus either indicate that estrous cycle of modern lactating dairy cows is greater than 18 to 24 d (Remnant et al., 2016) or is due to uncounted early embryonic losses before first pregnancy diagnosis (Wijma et al., 2016; Ricci et al., 2017; Domingues et al., 2024).

Delayed return to estrus could be partially attributable to prolonged LP unassociated with embryonic loss. This is difficult to assess because most data on cycle length are based on interservice intervals. In 14 herds, the incidence of normal (18–24 d) and prolonged (>24 d) interovulatory interval at the first postpartum cycle based on IMP4 was 41% and 30%, respectively (Tarekegn et al., 2019). A study in a single herd compared CL lifespan in cows inseminated but subsequently confirmed nonpregnant at 31 d versus cows receiving a placebo AI after estrus synchronization (Denis-Robichaud et al., 2024). Based on CL exams at d 17, 24, and 31, the odds of having a prolonged cycle were similar between groups. The prevalence of long cycle (estrus detection or CL regression by d 31) or very long cycle (no CL regression by d 31) was 20% and 36% in cows receiving AI, and 18% and 42% in cows receiving placebo AI, respectively. The high prevalence of prolonged cycles after placebo AI could be attributed, at least partially, to the frequency of CL examination if new ovulations occurring before or between examinations were not detected. Regardless, taken together, these studies suggest that a substantial proportion of cows may have prolonged cycles, contributing to delayed return to estrus that is unrelated to early pregnancy losses. These may occur due to alterations in ovarian physiology, leading to unexpected cycle lengths.

The cause of prolonged cycles is not fully understood but may be related to breed, lactation, and to reproductive tract disorders. In >2,600 cows, the length of the first postpartum LP was greater in Swedish Holstein (14 d) compared with Swedish Red (11 d) cows (Tarekegn et al., 2019). In >42,000 cows in 159 herds, the variation in interservice intervals was estimated to be 1% attributable to herd effects, 12% to cow-level variation, and 87% to different cycles within a cow (Remnant et al., 2015). Greater interservice intervals were associated with greater parity, greater 305-d milk yield, and greater days postpartum. Although the association between milk yield and interservice interval was very small in magnitude, the combination of factors suggests that a group of higher producing cows (as associated with greater parity), or cows at a higher producing stage of lactation, have prolonged estrous cycles. In that dataset, however, early pregnancy losses would contribute to estimates of extended interservice intervals. Others have also observed associations of greater milk yield around time of estrus with greater follicle and CL sizes, but lesser circulating concentrations of estradiol (**E2**) and P4 (Lopez et al., 2005). Milk yield per se is likely not physiologically linked to fertility, but the high metabolic clearance rate of circulating steroid hormones in high-producing cows could contribute to alterations in ovarian function due to reduced circulating E2 and P4 (Wiltbank et al., 2006).

Metabolic and physiological changes that high-producing dairy cows undergo during the transition period, especially when they are associated with health disorders, can affect reproductive function beyond the first postpartum ovulation (Bruinjé and LeBlanc, 2024). Opsomer et al. (2000) reported 20% of 448 cows with a prolonged LP (>20 d) before first AI. Among those, 50% had no detectable abnormalities, 2% had an ovarian cyst, and 48% had abnormal uterine contents. Cows diagnosed with metritis, abnormal vaginal discharge, or any clinical disease (milk fever, ketosis, mastitis, lameness, or displaced abomasum) in the first month of lactation had increased odds—by factors of 10, 4, and 3, respectively—of having a prolonged LP compared with healthy cows (Opsomer et al., 2000). Similarly, Ranasinghe et al. (2011) observed that 12% of 497 cows had a prolonged LP in the first 90 DIM, which was more likely to occur in multiparous than primiparous cows, in cows with earlier onset of cyclicity, and in cows with reproductive tract disease (dystocia, retained placenta, metritis, purulent vaginal discharge, or pyometra). These observational studies elucidate that clinical disease, particularly in the reproductive tract, is associated with prolonged estrous cycles.

To explore possible links between inflammatory disorders and ovarian cyclicity, we compared 139 cows that had markers of systemic inflammation (serum haptoglobin ≥0.8 g/L at wk 1 postpartum) and uterine inflammation (≥6% polymorphonuclear cells in endometrial cytology at 35 DIM) with 133 clinically healthy cows without indication of inflammation (Bruinjé et al., 2024). Cows with inflammatory disorders had 3.4-fold greater odds of having a prolonged LP (≥21 d) from 35 to 70 DIM than healthy cows (67% vs. 37% prevalence, respectively). They also had LP with lesser peak P4 concentration than healthy cows (6.9 vs. 8.2 ng/mL). Following repeated intrauterine administration of *Escherichia coli* LPS during the first 9 d of the estrous cycle in heifers, Lüttgenau et al. (2016) observed decreased CL blood flow, CL size, and plasma P4 concentrations compared with a control estrous cycle. They also reported increased plasma concentration of PGF_2α_ metabolite and increased mRNA expression of *TLR4* and prostaglandin E synthase in the CL. The decreased CL activity and increased plasma PGF_2α_ metabolite may point toward earlier rather than delayed luteolysis. But the increased expression of the luteotropic factor prostaglandin E synthase suggested that inflammation may dysregulate CL function and lifespan and could also result in extended LP. Furthermore, naturally occurring chronic uterine inflammation may result in different ovarian responses than experimental LPS-induced inflammation. Nonetheless, it seems that prolonged estrous cycles could be a consequence of reproductive tract inflammatory disorders at least shortly after the disease is diagnosed.

Whatever the causes of prolonged cycles or delayed returned to estrus, monitoring the dynamics of luteal activity with IMP4 can be used to optimize reproductive management. The characterization of P4 profiles such as LP length and P4 concentrations before and after AI could identify subgroups of cows with different predicted potential of P/AI. Since postpartum health is associated with reproductive function, information on transition cow health can be incorporated to improve prediction models. The information on IMP4 profiles can be used for selective breeding decisions, and to develop strategies for targeted hormonal interventions before, during, or after AI in cows with suboptimal P4 profiles to improve P/AI. Such strategies remain to be tested but could include (1) inducing luteolysis in noninseminated cows with extended LP, (2) inducing ovulation at estrus in cows with a “low-quality” estrus and expected lower P/AI, or (3) supplementing P4 or inducing formation of accessory CL in cows with suboptimal P4 concentrations. The IMP4 studies summarized here only included cows that ovulated (based on IMP4) after estrus, but did not describe cows that failed to ovulate and had cessation of cyclicity. There is a need of comprehensive studies comparing IMP4 with other estrus detection tools. Integrating data on estrus behavior, such as from activity monitors, could improve our understanding on physiological estrus that are not followed by behavioral estrus, and be used to optimize prediction of P/AI and reproductive tactics. Furthermore, the economics of adopting IMP4 and the possibility to scale its use to large dairies should be investigated. With a growing number of herds and countries adopting IMP4 monitoring, there will likely be large amounts of data that could be valuable to better understand the estrous cycle of modern high-producing cows to optimize reproductive management and performance. The IMP4 technology was able to detect 85% of cows that were not pregnant at 55 d after AI returning to estrus before 30 d, which is the typical time of first pregnancy diagnosis in many dairies. This highlights the potential to optimize the early identification of nonpregnant cows for timely reinsemination.
